# Exercise Therapy Downregulates the Overexpression of TLR4, TLR2, MyD88 and NF-κB after Cerebral Ischemia in Rats

**DOI:** 10.3390/ijms14023718

**Published:** 2013-02-07

**Authors:** Yuewen Ma, Man He, Lin Qiang

**Affiliations:** Department of Rehabilitation Medicine, the First Affiliated Hospital, China Medical University, Shenyang 110001, Liaoning, China; E-Mails: xiaopanger1984@sina.com (M.H.); qianglin@yeah.net (L.Q.)

**Keywords:** neuroprotection, cerebral ischemia, TLR2, TLR4, exercise, NF-κB, MyD88

## Abstract

Toll-like receptor 2 (TLR2) and Toll-like receptor 4 (TLR4) are considered to mediate the inflammatory reaction of cerebral ischemia injury, and exercise can inhibit the activity of the Toll-like receptor signaling pathway in the peripheral blood of humans. Although physical exercise has been demonstrated to be neuroprotective in both clinical and laboratory settings, the underlying mechanism remains unclear. To clarify this critical issue, this study investigated the effects of treadmill training on the recovery of neurological function and the expression of TLR2 and TLR4 and their main downstream targets, nuclear factor-kappaB (NF-κB) and myeloid differentiation factor 88 (MyD88), in the ischemic rat brain after middle cerebral artery occlusion-reperfusion (MCAo/R). Rats were divided into seven groups: sham control without MCAo/R and five, nine and 16 days post-ischemic exercise or non-exercise. The neurological function and infarct volume were measured, and reverse transcription polymerase chain reaction (RT-PCR) and Western blotting were used to detect the expression of TLR2, TLR4, NF-κB and MyD88 in ischemic brain tissue. The results indicated that treadmill training promoted functional recovery and reduced the overexpression of TLR2, TLR4, NF-κB and MyD88 in rat brain tissue after ischemia, a finding that may have implications for understanding the mechanism of exercise therapy after brain ischemia and indicating new therapeutic strategies for the pharmacological modulation of TLR signaling.

## 1. Introduction

Recent experimental and clinical studies have highlighted a complex role for the immune system in the pathophysiological changes that occur after acute stroke. Sensors of the innate immune system, such as Toll-like receptors (TLRs), and effectors, such as innate immune cells and the lectin pathway of complement activation, are activated by brain ischemia and tissue damage, leading to an amplification of the inflammatory cascade. TLRs are evolutionarily conserved pattern-recognition receptors that recognize pathogen-associated molecular patterns and endogenous danger-associated molecular patterns released as a result of tissue damage. TLR2 and TLR4 were recently shown to contribute to ischemic brain damage in mice, potentially by activating proapoptotic pathways and the release of proinflammatory cytokines [1–8], particularly during the acute phase of ischemic stroke [9].

Both TLR2 and TLR4 function in the MyD88-dependent pathway to activate NF-κB, which induces the expression of proinflammatory genes, inflammatory cytokines and adhesion molecules and the activation of adaptive immunity [10]. In unstimulated cells, NF-κB resides in the cytoplasm as a complex with inhibitory IκB proteins that mask their nuclear localization signal. Upon cell activation, IκB is phosphorylated and proteolytically degraded, resulting in the translocation of NF-κB to the nucleus [11]. NF-κB consists of five subunits: c-Rel, RelB, p65 (RelA), p105 (NF-κB1) and p100 (NF-κB2). NF-κB is activated in the ischemic lesion of a rodent model of focal cerebral ischemia, as determined by the nuclear translocation of NF-κB in neurons and the enhanced DNA binding of NF-κB subunits p65 and p50 [12]. In p50^−/−^ mice, permanent middle cerebral artery occlusion (MCAo) resulted in 41% smaller infarcts compared with wild-type controls [13]. Accumulating evidence supports the hypothesis that elevated NF-κB contributes to ischemia-induced neurological injury [14,15], suggesting that NF-κB inhibition may represent a treatment target in ischemic stroke.

Exercise is a well-known component of stroke rehabilitation programs. Exercise studies in ischemic rat models have demonstrated that exercise can improve the post-ischemic functional recovery of behavior and structural alterations in the brain [16–19]. Physical rehabilitation that is initiated early confers marked neuroprotection against experimental stroke by attenuating pro-inflammatory reactions, brain edema, blood-brain barrier damage and cognitive and behavioral deficits [20]. Studies have shown that movement can modulate the internal inflammatory response through the TLR4 and TLR2 signaling pathway in peripheral blood [21–23] and reduce TLR4 in muscle [24]. Although the beneficial effects on brain function have been confirmed, the detailed mechanisms responsible for this exercise-induced neuroprotection remain poorly understood.

Accordingly, new approaches to explore the mechanism of exercise therapy and new treatments are desperately needed. One such approach involves understanding the role of the pathways leading to inflammation, such as the TLR signaling pathway, in controlling the brain’s response to exercise after ischemia. Our objective in this study was to show that treadmill training after cerebral infarction reduces in TLR4, TLR2, MyD88 and NF-κB expression in rat brain tissue, effects that are correlated with better functional recovery.

## 2. Results and Discussion

### 2.1. Exercise Promotes Neurological Function Recovery after MCAo/R

**Figure 1:**
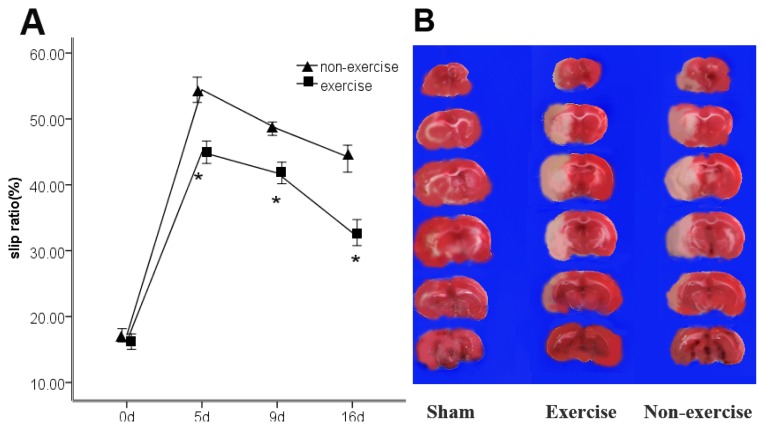
(**A**) Sensorimotor recovery in rats in the exercise group and the non-exercise group is shown as the slip ratio at five days, nine days and 16 days after middle cerebral artery occlusion-reperfusion (MCAo/R). The mean ± s.e.m. is presented. * Different from the operation control group (*p* < 0.05); (**B**) This photograph shows a representative cerebral infarct of the brain slices in the sham, exercise and non-exercise groups at 16 days. The pale region is the infarct brain tissue, and the red region is normal tissue. The infarct volume of five rats in the sham group was zero. There was no statistically significant difference between the indicated exercise group and non-exercise group.

As shown in Figure 1, ischemia caused hindlimb impairment in rats. There was a significant difference in the slip ratio of the impaired hindlimb between the non-exercise with stroke group and exercise with stroke group, suggesting that treadmill training significantly improved beam-walking performance (Figure 1A).

### 2.2. Exercise Has No Obvious Effect on the Infarct Volume

No infarction was observed in the sham-operated group, whereas extensive lesions developed in the MCAo/R group. There was no significant difference between the exercise group (43.7% ± 3.8%) and non-exercise group (45.6% ± 2.3%) after two weeks of treadmill training (Figure 1B).

### 2.3. Decreased *TLR2*, *TLR4*, *MyD88* and *NF-κB* Gene Expression after Exercise

**Figure 2:**
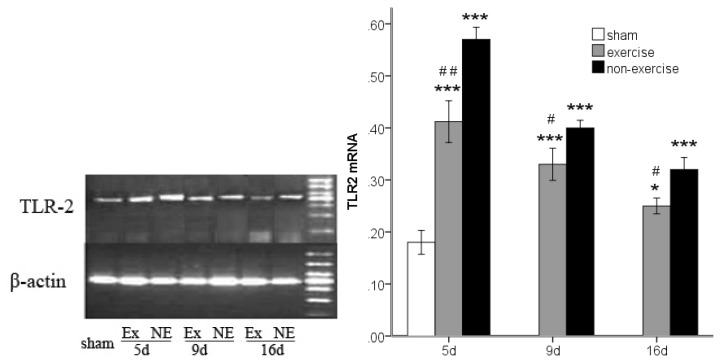
Toll-like receptor 2 (TLR2) mRNA expression in peripheral ischemia over time after MCAo/R injury. The experimental groups are represented by the following abbreviations: sham group (sham), exercise group (Ex) and non-exercise group (NE). The data (*n* = 5) are the mean ± s.e.m. *p < 0.05, ***p < 0.001 compared to the sham group; ^#^*p* < 0.05, ^##^*p* < 0.01 compared to the non-exercise group.

**Figure 3:**
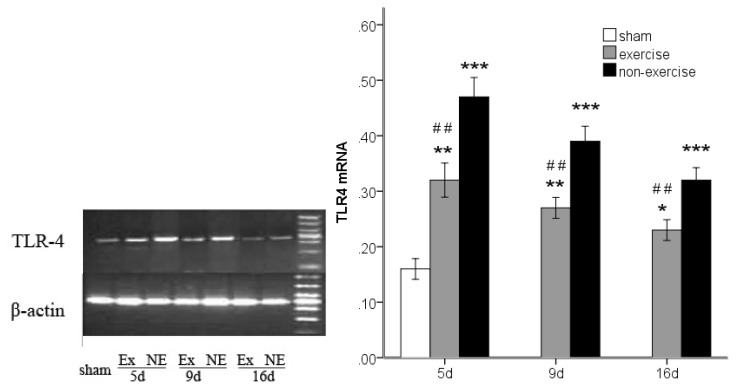
TLR4 mRNA expression in peripheral ischemia over time after MCAo/R injury. The experimental groups are represented by the following abbreviations: sham group (sham), exercise group (Ex) and non-exercise group (NE). The data (*n* = 5) are the mean ± s.e.m. *p < 0.05, **p < 0.01, ***p < 0.001 compared to the sham group; ^#^*p* < 0.05, ^##^*p* < 0.01 compared to the non-exercise group.

**Figure 4:**
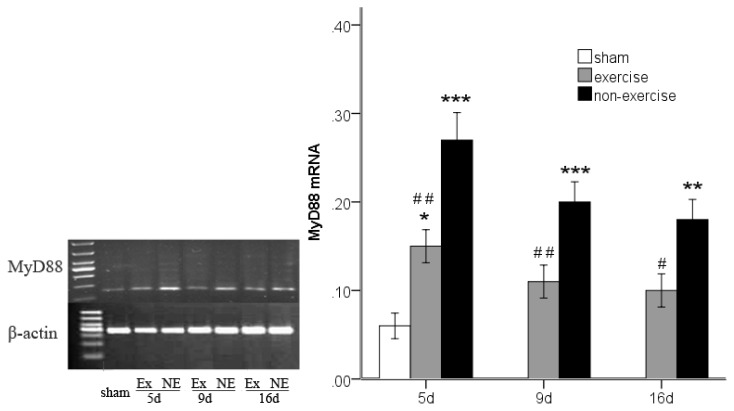
MyD88 mRNA expression in peripheral ischemia over time after MCAo/R injury. The experimental groups are represented by the following abbreviations: sham group (sham), exercise group (Ex) and non-exercise group (NE). The data (*n* = 5) are represented as the mean ± s.e.m. *p < 0.05, **p < 0.01, ***p < 0.001 compared to the sham group; ^#^*p* < 0.05, ^##^*p* < 0.01 compared to the non-exercise group.

**Figure 5:**
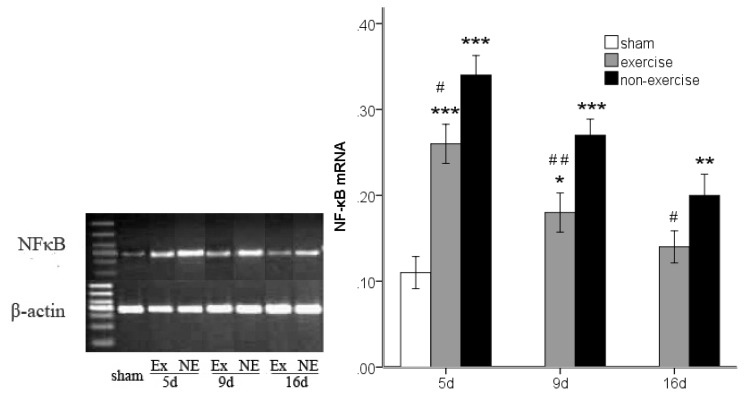
NF-κB mRNA expression in peripheral ischemia over time after MCAo/R injury. The experimental groups are represented by the following abbreviations: sham group (sham), exercise group (Ex) and non-exercise group (NE). The data (*n* = 5) are presented as the mean ± s.e.m. *p < 0.05, **p < 0.01, ***p < 0.001 compared to the sham group; ^#^*p* < 0.05, ^##^*p* < 0.01 compared to the non-exercise group.

*TLR2*, *TLR4*, *MyD88* and *NF-κB* gene expression was determined by measuring the messenger RNA levels of the genes using reverse transcription polymerase chain reaction (RT-PCR). An analysis of variance showed that MCAo/R lead to notably higher levels of all of these markers compared to the sham group (*p* < 0.001). However, after five, nine and 16 days of reperfusion in rats subjected to MCAo/R, a significant (*p* < 0.05) decrease in the messenger RNA of all these markers was found in the exercise group compared to the non-exercise control (Figures 2, 3, 4 and 5). The difference in MyD88 between the sham group and exercise groups was not significant at postoperative day nine (*p* = 0.56) and 16 (*p* = 0.16) and was the same as NF-κB at postoperative day 16 (*p* = 0.27). The treadmill training may explain the long-term declines of these markers to a nearly normal level.

### 2.4. Exercise Inhibits MCAo/R-Induced TLR2, TLR4 and NF-κB p65 Activation

**Figure 6:**
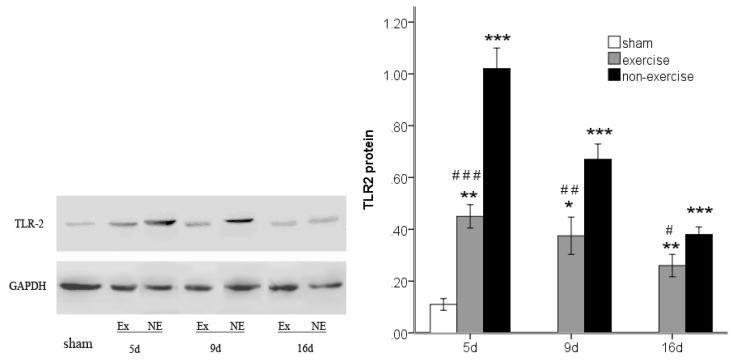
TLR2 protein expression in peripheral ischemia over time after MCAo/R injury. The experimental groups are represented by abbreviations as follows: sham group (sham), exercise group (Ex) and non-exercise group (NE). The data (*n* = 5) are the mean ± s.e.m. *p < 0.05, **p < 0.01, ***p < 0.001 compared to the sham group; ^#^*p* < 0.05, ^##^*p* < 0.01, ^###^*p* < 0.001 compared to the non-exercise group.

**Figure 7:**
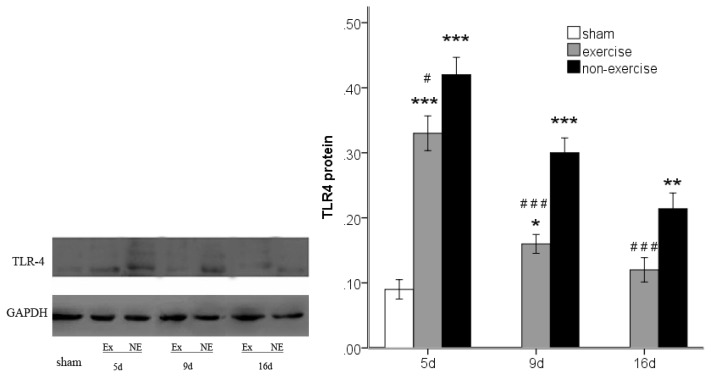
TLR4 protein expression in peripheral ischemia over time after MCAo/R injury. The experimental groups are represented by the following abbreviations: sham group (sham), exercise group (Ex) and non-exercise group (NE). The data (*n* = 5) are the mean ± s.e.m. *p < 0.05, **p < 0.01, ***p < 0.001 compared to the sham group; ^#^*p* < 0.05, ^###^*p* < 0.001 compared to the non-exercise group.

**Figure 8:**
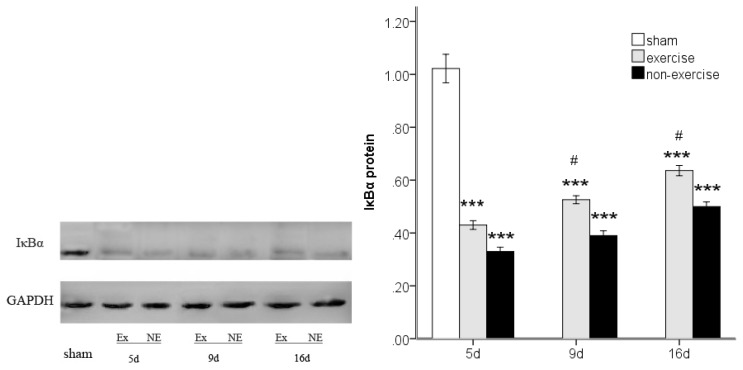
The changes in cytosolic IκBα in peripheral ischemia over time after MCAo/R injury. The experimental groups are represented by the following abbreviations: sham group (sham), exercise group (Ex) and non-exercise group (NE). The data (*n* = 5) are the mean ± s.e.m. ***p < 0.001 compared to the sham group; ^#^*p* < 0.05 compared to the non-exercise group.

**Figure 9:**
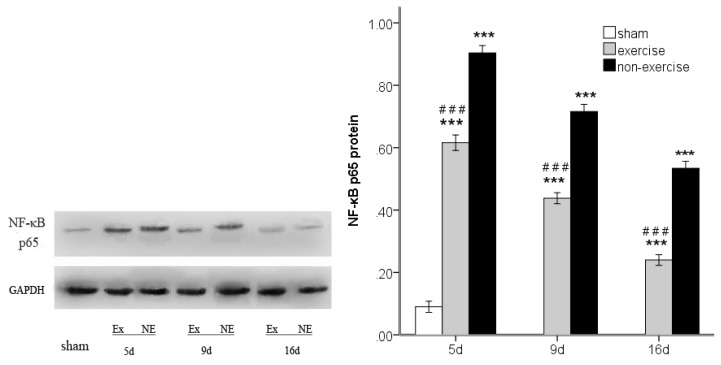
The changes in nuclear NF-κB p65 in peripheral ischemia over time after MCAo/R injury. The experimental groups are represented by the following abbreviations: sham group (sham), exercise group (Ex) and non-exercise group (NE). The data (*n* = 5) are the mean ± s.e.m. ***p < 0.001 compared to the sham group; ^###^*p* < 0.001 compared to the non-exercise group.

To investigate TLR2/4-mediated pro-inflammatory responses, we investigated the protein expression of TLR2 and TLR4 and the downstream transcription factor NF-κB p65 by Western blotting. MCAo/R significantly (*p* < 0.001) increased TLR2 and TLR4 expression compared to the sham group at postoperative days five, nine and 16, and the expression of these proteins decreased over time (Figures 6 and 7). Both TLR2 and TLR4 exhibited lower expression compared to the MCAo/R control group (*p* < 0.001). As the degradation of inhibitory kappa B alpha (IκBα) proteins is an essential step for NF-κB p65 activation [25], we further examined the effect of exercise on MCAo-induced IκBα degradation. We found that the cytosolic protein levels of IκBα, an upstream mediator in the NF-κB pathway, were markedly decreased in the ischemic cortex after MCAo/R (Figure 8); however, this decrease was attenuated by exercise (*p* < 0.05). The results also showed that NF-κB p65 expression was dramatically upregulated in the ischemic cortex after MCAo/R (Figure 9); exercise therapy significantly reduced the protein level of NF-κB p65 after MCAo/R in the exercise group (*p* < 0.01) *versus* the non-exercise group (Figure 9). In agreement with the RT-PCR results (Figures 2, 3 and 5), a Western blot (Figures 6–9) analysis showed that exercise significantly suppressed the TLR2, TLR4 and NF-κB p65 levels in the exercise group (*p* < 0.05).

### 2.5. Discussion

In this paper, we sought to describe the exercise therapy-induced reprogrammed TLR2/4 signaling events involved in neuroprotection against ischemic injury. We demonstrated an upregulation of TLR2, TLR4 and NF-κB p65 protein and *TLR2*, *TLR4*, *NF-κB* and *MyD88* mRNA in a standard experimental stroke model. After performing treadmill training in rats, we further investigated the time course of TLR2/4 signaling. Our data showed that exercise therapy promotes neurological recovery by attenuating the overexpression of TLR2, TLR4, MyD88 and NF-κB, thus providing neuroprotection. Previous reports have demonstrated that there is a nonsignificant trend toward smaller lesions when animals are trained only after ischemia [26], and our experimental results confirmed this view.

Exercise training has been well-established by prior studies to protect against ischemia-induced brain injury and is particularly helpful in recovery following brain ischemia [16,18,19,27–29]. The mechanisms underlying this neuroprotection may include the attenuation of pro-inflammatory reactions, brain edema, blood-brain barrier damage and cognitive and behavioral deficits [20]. Motor function and striatal angiogenesis were reported to change significantly in treadmill training groups compared to the control group [30]. The present study suggests an additional mechanism underlying the neuroprotective effect of early exercise: regulation of the TLR2/4 signaling pathway.

Post-ischemic inflammation is an essential step in the progression of brain ischemia-reperfusion injury. The brain is a sterile organ, but injury-induced inflammation is mostly dependent on TLR2/4. Along with MyD88, the main downstream adaptor, TLR2/4 mediates pro-inflammatory responses through NF-κB and other sensors, which induces the upregulation of pro-inflammatory factors. The expression of NF-κB in the rat brain cortex was markedly higher after MCAo/R at 24 h and lasted up to 48 h [31]. In human specimens of infarcted brains, NF-κB was found to be activated in neurons in the penumbral areas during the first two days of human stroke; intense cytoplasmic NF-κB staining was noted in the neurons on the first day, whereas nuclear staining dominated neuronal NF-κB expression by the second day [13]. In our study, we found that NF-κB p65 expression was still high in the nucleus at five days after MCAo/R. Some endogenous TLR ligands, high-mobility group box 1 (HMGB1) and, in particular, peroxiredoxin family proteins have been implicated in the activation of infiltrating macrophages and inflammatory cytokine expression in these cells. Following macrophage activation, T-lymphocytes infiltrate the ischemic brain and regulate delayed-phase inflammation [32]. The activation of the adaptive arm of the immune system, mediated by lymphocyte populations, including Tand B-cells, regulatory T-cells and γδT-cells, in response to stroke can lead to deleterious antigen-specific autoreactive responses, but can also have cytoprotective effects. An increased incidence of infection is observed after acute stroke and might result from the activation of long-distance feedback loops between the central nervous system and peripheral immune organs, which are thought to play a role in stroke-induced immunodepression [33,34].

The amount of brain damage and neurological deficits caused by a stroke was significantly lower in mice deficient in TLR2 or TLR4 compared with wild-type control mice [5]. A proapoptotic signaling pathway for TLR2 and TLR4 in neurons may render them vulnerable to ischemic death. The TLR2 protein, which is mainly expressed in microglia, selected endothelial cells, neurons and astrocytes in the post-ischemic brain tissue, induced the production of TLR2-related genes with pro-inflammatory and pro-apoptotic capabilities [6]. However, Hua F. *et al.* found that TLR4 contributes to cerebral ischemia/reperfusion injury, whereas TLR2 appears to be neuroprotective by enhancing the activation of protective signaling in response to cerebral ischemia/reperfusion [7]. Numerous preconditioning stimuli can lead to TLR activation, an event that occurs prior to ischemia and ultimately leads to TLR reprogramming [9,35–37]. Such reprogramming leads to the suppressed expression of pro-inflammatory molecules and the enhanced expression of numerous anti-inflammatory mediators, which collectively confer robust neuroprotection. Thus, the genomic reprogramming of TLR signaling may be a unifying principle of tolerance to cerebral ischemia.

Exercise has been shown to inhibit the expression of TLR2/4 on the surface of monocytes in the peripheral blood of volunteers [22,38]. Moreover, through long-term observation, McFarlin B and Flynn M found that the low TLR4 expression induced by exercise can improve the body’s chronic inflammatory state [38]. Previous research has indicated that pre-ischemic exercise can reduce the ischemic infarct volume by regulating expression of TLR4 in brain tissue [9]. However, a relationship between exercise therapy post-cerebral infarction and TLRs has not been reported. Our experiment demonstrated the direct intervention of the TLR2 and TLR4 pathway cascade after cerebral ischemia by treadmill training, with an improvement in the neurological score.

We used tapered/ledged beam tests to evaluate the influence of treadmill training on the expression of TLR4, TLR2, MyD88 and NF-κB in focal cerebral infarcts in rats. Our results showed that exercise induced an important suppression of the TLR2/4 signaling pathway, as evidenced by reductions in an early step of this pathway, *i.e.*, TLR4/MyD88 interactions. Indeed, we found that MCAo/R caused NF-κB activation, as revealed by the total NF-κB mRNA expression and nuclear translocation of the NF-κB subunit p65 at all the time points studied and by a decrease in the expression of cytosolic IκBα. Furthermore, treadmill training in rats significantly reduced NF-κB p65 activation at the time points examined when compared with the non-exercised rats. Ample evidence indicates that NF-κB is activated in cerebral ischemia and reperfusion (I/R), particularly in neurons [39,40]. Our data strongly support the theory that TLR2/4 signaling is implicated in exercise therapy neuroprotection after cerebral ischemia through the inhibition of NF-κB activation. These alterations are associated with a significant improvement in the behavioral outcome. We hypothesize that the signal transduction pathway was activated after endogenous activators were recognized by TLR2/4, leading to the recruitment of MyD88, activation of NF-κB and subsequent release of proinflammatory cytokines, resulting in tissue injury. By decreasing the expression of the TLR2/4 gene and protein, exercise therapy can reduce the expression of MyD88 and NF-κB, which can inhibit inflammatory injury, weaken cerebral edema and provide neuroprotection against subsequent cerebral ischemic injury. The mechanism through which TLR2/4 and a series of ensuing inflammatory factors regulate the immune system require further research. In addition, it remains unclear whether the TLR2 and TLR4 we tested were localized in residual neurons or infiltrated cells from the peripheral immune system [9]. Although the potential mechanisms underlying the exercise therapy neuroprotection are unknown, our study showed that exercise therapy may ameliorate the inflammation by reducing the expression of TLR4, TLR2, MyD88 and NF-κB. These findings suggest a potential treatment of cerebral ischemic injury through the reduction of TLR2/4 activation.

## 3. Materials and Methods

### 3.1. Animal Preparation

Adult male Wister rats (250–350 g, clean animals, Animal Department of China Medical University, Liaoning, China) were randomly assigned to three groups: sham operation group (sham group) (*n* = 10), MCAo/R and exercise group (exercise group) (*n* = 20) and MCAo/R control group (non-exercise group) (*n* = 20). The exercise group and non-exercise group were divided into three sub-groups of different observation time points after surgery: 5 days and 9 days for 5 rats in each subgroup and 16 days for 10 rats in each subgroup. The animals in each group were housed and bred in the same animal care facility under a 12-hour light/dark cycle throughout the study. The rats had free access to food and water. All experiments were conducted in accordance with the Guidelines for Animal Experimentation of the Institutional Animal Care and Use Committee of China Medical University. All efforts were made to minimize any suffering and reduce the total number of animals used.

### 3.2. Middle Cerebral Artery Occlusion-Reperfusion (MCAo/R) Model

We induced transient focal cerebral ischemia, which was allowed to reperfuse after 2 h, through intraluminal middle cerebral artery occlusion (MCAo) with a monofilament according to the modified suture-occluded method of Zea Longa [41]. Briefly, the rats were anesthetized with 10% chloral hydrate (350 mg/kg, i.p.). The cervical vessels were exposed through a median ventral cervical incision in the skin, and the left common cervical artery (CCA), left external carotid artery (ECA) and left internal carotid artery (ICA) were isolated. The ECA and CCA were tied close to the origin. The 3-0 silk suture was tied loosely around the ICA, and a micro-vascular clip was placed distally across the ICA. A puncture was performed at the ICA-ECA bifurcation and a polyester monofilament thread (body diameter, 0.265 mm; head diameter, 0.34 ± 0.02 mm), with its tip rounded by heating, was introduced into the ICA. The suture around the ICA (and the polyester thread) was tightened, and the microvascular clip was removed. The polyester thread was then gently advanced into the ICA lumen for 18–20 mm until it reached and occluded the ostium of the left MCA. At 2 h after the occlusion, the filament was withdrawn to allow reperfusion. The sham-operated rats underwent the same surgical procedure, except that the polyester thread was advanced into the ICA for 9 mm. The rectal temperature was maintained at 37.0–37.5 °C with a heating pad throughout the surgical procedures. The rats were given 20,000 U gentamicin (i.p.) within 3 days after model establishment. The neurologic deficits were scored using a modified scoring system based on the system developed by Longa *et al.* [41]: 0, no deficits; 1, difficulty in fully extending the contralateral forelimb; 2, unable to extend the contralateral forelimb; 3, mild circling to the contralateral side; 4, severe circling; and 5, falling to the contralateral side. Three animals died after MCAo/R and the same number of animals was supplemented. Rats with a score of 2–4 were included in the experimental group.

### 3.3. Behavioral Outcome Measures in Rats after Focal Cerebral Ischemia

The tapered/ledged beam tests selected for the study are sensitive for the detection of long-term impairment in sensorimotor functions. The issue of analyzing the recovery of original function *versus* learned motor compensation was resolved by having ledges along both sides of the beam, providing a crutch on which the animal can place the forelimbs or hindlimbs that slip off the upper beam. Compensatory adjustment in posture or weight bearing in the nonimpaired limbs is unnecessary when the animal traverses the beam. The animals were tested before the operation and on postoperative days 5, 9 and 16. All behavioral analyses were performed in a blinded manner. The rats were pretrained for 3 days to traverse the beam before ischemia induction. The beam-walking apparatus consisted of a tapered beam with underhanging ledges on each side to permit foot faults without falling. The end of the beam was connected to a black box (20.5 × 25 × 25 cm^3^) with a platform at the starting point. A bright light was placed above the start point to motivate the rats to traverse the beam. Each rat’s performance was videotaped and subsequently analyzed by calculating the slip ratio of the impaired (contralateral to lesion) hindlimb: More slips indicated a greater degree of impairment. Steps onto the ledge were scored as a full slip, and a half slip was scored if the limb touched the side of the beam. The slip ratio was calculated as follows: ((number of full slips + 0.5 × number of half slips)/(number of total steps)) × 100%. The mean of three trials was used for the statistical analysis.

### 3.4. Physical Exercise

With 3 days of adaptability to treadmill training before surgery, the treadmill training-treated animals were exercised on a four-lane treadmill (JX-240 treadmill, Xuzhou Fitness Equipment Company, Xuzhou, China) at 3 days after surgery at a speed of 12 m/min for 30 min each day, 5 days a week. The non-exercised controls and the exercised animals were housed in standard cages in small groups (*n* = 5) for equal time periods (exercised for 3 days, 7 days and 2 weeks, respectively).

### 3.5. Measurement of Cerebral Infarction Volume

Five rats from the sham-operated group, five rats from the 14-day exercise group (16 days post stroke) and five rats from the 16-day post-stroke non-exercise group, respectively, were used. The animals were euthanized with chloral hydrate and the brains were collected quickly and placed at −20 °C for 30 min. The brain tissue was cut into 6 coronal Sections 2 mm thick and stained with a 2% solution of triphenyltetrazolium chloride (TTC) in phosphate-buffered saline (PBS) at a temperature of 37 °C for 20 min, followed by 4% paraformaldehyde buffer for fixation. The stained sections were photographed, and the digital images were analyzed using NIH image analyzer software [42]. The infarct volume was calculated as the difference between the volume of the contralateral hemisphere and the volume of the TTC-stained portion (non-ischemic) of the ipsilateral hemisphere in each rat. The total infarction volume was calculated as the sum of the area of the brain infarction multiplied by the thickness of each section (3 mm). The lesion volumes were calculated by the following formula: {[total infarct volume − (the volume of the intact ipsilateral hemisphere − the volume of the intact contralateral hemisphere)]/contralateral hemisphere volume} × 100%. This indirect measure corrects for edema in the total infarct volume.

### 3.6. TLR2, TLR4, MyD88 and NF-κB p65 Messenger RNA Expression

At the relevant time points, the rats were sacrificed by decapitation and the ipsilateral cortical tissues between the anterior and posterior fontanel were dissected and frozen. The brain tissues were equally divided into 2 parts: The anterior parts were used for extracting RNA and the posterior for Western blot analysis.

The RT-PCR (reverse transcription polymerase chain reaction) technique was used to determine the expression of genes encoding TLR2, TLR4, MyD88 and NF-κB. Total RNA was isolated from the sham, non-exercise and exercise groups using a reverse transcription kit (RNAiso™ PLUS, TAKARA, city, country). We obtained cDNA by reverse transcription, which was performed using a reverse transcription kit (TAKARA), according to the manufacturer’s protocol. The amplification was performed as follows: for TLR4, MyD88 and β-actin, 35 cycles of predenaturation at 94 °C for 2 min, denaturation at 94 °C for 30 s, annealing at 58 °C for 30 s and elongation at 72 °C for 30 s; and for TLR2 and NF-κB, 35 cycles of predenaturation at 94 °C for 2 min, denaturation at 95 °C for 30 s, annealing at 56 °C for 30 s and elongation at 72 °C for 30 s. The PCR primers were as follows: TLR4 (356 bp), 5′-GCCGGAAAGTTATTGTGGTGGT-3′ (forward) and 5′-ATGGGTTTTAGGCGCAGA GTTT-3′ (reverse); TLR2 (93 bp), 5′-TATCAGTCCCAAAGTCTAAAGTCG-3′ (forward) and 5′-CTACCTCCGACAGTTCCAAGATG-3′ (reverse); NF-κB (424 bp), 5′-GCGCATCCAGACCAA CAATAA-3′ (forward) and 5′-GCCGAAGCTGCATGGACACT-3′ (reverse); Myd88 (398 bp), 5′-CAACCAGCAGAAACAGGAGTCT-3′ (forward), 5′-ATTGGGGCAGTAGCAGATGAAG-3′ (reverse); and β-actin (372 bp), 5′-GCCATGTACGTAGCCATCCA-3′ (forward) and 5′-GAACC GCTCATTGCCGATAG-3′ (reverse). The amplified samples were subjected to electrophoresis through 2% agarose gel. The fluorescence intensity of each band was scanned and quantified using NIH image software. The levels of TLR4, TLR2, MyD88 and NF-κB mRNA were normalized against those of β-actin mRNA.

### 3.7. Western Blot Analysis

Brain areas used for the Western blot analysis were collected and homogenized as previously described [43]. NF-κB was examined in the cytosolic and nuclear extracts, which were prepared as previously described [43]. Equal amounts of protein from brain tissue in the middle cerebral artery-supplied region were separated on 10% sodium dodecyl sulfate-polyacrylamide gels for 35 min at 200 V and transferred onto a 0.2 μm polyvinylidene fluoride (PVDF) membrane (Thermo Scientific, city, country) at 70 V for 2 h. The membranes were blocked for 2 h at room temperature with 5% skim milk and washed 3 times with Tris-buffered saline-Tween buffer, for 10 min each time. The membranes were then incubated with specific primary antibodies against TLR4 (Abcam, Cambridge, MA, USA, 1:1000), TLR2 (Abcam, Cambridge, MA, USA, 1:1000), NF-κB p65 (Santa Cruz Biotechnology, Santa Cruz, CA, USA, 1:500) and IκBa (Santa Cruz Biotechnology, Santa Cruz, CA, USA, 1:500) overnight at 4 °C. The membranes were washed three times with Tris-buffered saline-Tween buffer, incubated for 2 h with rabbit anti-rat horseradish peroxidase (HRP) (1:5000; Zhongshanjinqiao, Beijing, China) and washed three times with PBS-Tween. The immunoreactive bands were visualized with the electrochemiluminescence (ECL) system (Baoxin Biotec, Shenyang, China), and a microplate reader was applied to measure optical density at 540 nm and generate a standard curve. Glyceraldehyde 3-phosphate dehydrogenase (GAPDH) was used as a loading control to confirm the equality of total added protein.

### 3.8. Statistical Analysis

SPSS software (Version 20.0) was used for all the statistical analyses. The beam-walking data for the overall group effect were analyzed using an analysis of variance (ANOVA) for repeated measures. For the gene and protein expression data, a one-way ANOVA was used to identify differences between the groups, followed by a post-LSD (least significant difference) test. All correlations are presented as Pearson’s two-way correlations. The level of statistical significance was *p* < 0.05 throughout this study.

## 4. Conclusions

Our study confirmed that treadmill training protected the brain from the damage caused by MCAo, an effect that may occur through the downregulation of TLR4, TLR2, MyD88 and NF-κB expression. The elevated expression of TLR4, TLR2, MyD88 and NF-κB is thought to be consistent with ischemia-evoked neuron injury and death through an inflammatory mechanism. The TLR4, TLR2, MyD88 and NF-κB pathways may be strategic targets for cerebral ischemic therapies. The combination of exercise therapy and modulators of these targets will produce a better recovery of cerebral infarction.
